# Tumor-Associated Neutrophils and Desmoplastic Reaction in Breast Cancer Microenvironment: Association with Tumor Grade and Clinicopathological Features

**DOI:** 10.3390/cancers18030406

**Published:** 2026-01-27

**Authors:** Stavroula Papadopoulou, Vasiliki Michou, Arsenios Tsiotsias, Maria Tzitiridou-Chatzopoulou, Panagiotis Eskitzis

**Affiliations:** Department of Midwifery, School of Healthcare Sciences, University of Western Macedonia, Keptse, 50200 Ptolemaida, Greece; staupapadop@yahoo.gr (S.P.); atsiotsias@uowm.gr (A.T.); mtzitiridou@uowm.gr (M.T.-C.); peskitzis@uowm.gr (P.E.)

**Keywords:** breast cancer, tumor microenvironment, tumor-associated neutrophils (TANs), desmoplastic reaction, tumor grade, clinicopathological features, prognosis, extracellular matrix

## Abstract

Background: The tumor microenvironment (TME), composed of diverse immune and stromal cells, plays a key role in cancer progression. Among its components, tumor-associated neutrophils (TANs) and the desmoplastic reaction (DR) have emerged as important modulators of tumor behavior. While each has been extensively studied, their interrelationship and association with tumor grade and clinicopathological parameters remain unclear. Aim: This hypothesis-generating study aimed to explore the relationship between the presence of TANs, various types of DR, the grade of tumor malignancy, and other fundamental clinicopathological characteristics commonly studied in daily clinical practice. Materials and Methods: The study included a cohort of 65 cancer patients (N = 65). The average number of TANs was recorded. In hematoxylin and eosin (H&E)-stained sections, “hot spots” representing areas with the highest neutrophil density were first identified. The tumor-associated polymorphonuclear neutrophils were then counted in ten consecutive high-power fields (HPFs). In the same specimens, the DR was assessed and classified according to stromal texture. Results: TANs did not follow a normal distribution across any clinicopathological category (*p* < 0.05). Significant differences in TAN levels were observed among DR types (Kruskal–Wallis H = 9.890, *p* = 0.007), with higher counts in myxoid compared to mature stroma (Mean Rank = 41.58 vs. 24.80, *p* = 0.006). TAN levels also varied significantly with tumor grade (H = 22.384, *p* < 0.001), increasing from Grade 1 to Grade 3 (*p* < 0.013–0.001). Higher TAN counts were associated with cellular erythroblastic oncogene B2 (c-erbB2) positivity (H = 6.547, *p* = 0.038), perineural invasion (Mann–Whitney U = 179.5, *p* < 0.001), and ER/PR negativity (*p* = 0.016 and *p* = 0.044, respectively). No significant association was found with necrosis (*p* = 0.083). A near-significant relationship was identified between DR type and tumor differentiation grade (χ^2^ = 9.448, *p* = 0.051), with mature stroma most common in Grade 1 tumors, keloid-like stroma in Grade 2, and myxoid stroma in Grade 3. Conclusions: High TAN levels were linked to aggressive tumor features and specific DR patterns. The association of myxoid stroma with elevated TAN infiltration may reflect a highly aggressive TME. These preliminary results warrant validation in larger, prospective studies.

## Simple Summary

Cancer growth and spread are strongly influenced by the surrounding tissue, known as the tumor microenvironment. This environment contains immune cells and connective tissue that can either slow down or promote tumor progression. In this study, we focused on a specific type of immune cell, neutrophils, and on changes in the connective tissue surrounding tumors, known as the desmoplastic reaction. We examined tissue samples from 65 cancer patients to understand how these features relate to tumor aggressiveness and common clinical characteristics. We found that higher numbers of neutrophils were associated with more aggressive tumors and specific connective tissue patterns. In particular, tumors with loose, myxoid connective tissue showed increased neutrophil infiltration. These findings suggest that interactions between immune cells and connective tissue contribute to tumor behavior and help improve future tumor evaluation and risk assessment.

## 1. Introduction

Breast cancer remains one of the most prevalent malignancies and continues to be a leading cause of cancer-related mortality worldwide [1]. In 2022 alone, an estimated 2.3 million women were diagnosed with the disease, and approximately 670,000 deaths were recorded globally [2]. This type of cancer can affect women of any age after puberty, with incidence rates increasing as they get older, and it is seen across different populations worldwide [1,2]. In recent years, scientific focus has shifted from cancer cells alone to the tumor microenvironment (TME), which is increasingly recognized for its critical role in tumorigenesis, disease progression, metastasis, and response to therapy [3,4,5,6,7,8,9,10,11,12,13,14,15,16]. The TME is a complex biological entity composed of diverse cellular and non-cellular components, including immune cells, fibroblasts, endothelial cells, signaling molecules, and the extracellular matrix (ECM) [3,4,5,6,7,8,9,10,11,12,13,14,15,16,17]. Among these components, the cells of the immune system and their associated elements are increasingly recognized as vital contributors to the development and progression of cancer [18].

In addition, recent data indicate that the interaction between malignant cells and leukocytes significantly influences the effectiveness of chemotherapy [19]. Numerous recent studies have focused on neutrophils, and in particular TANs [10,11,12,14,15,20,21,22,23,24], which exhibit a remarkable property known as “plasticity,” defined as the capacity to either promote or inhibit tumor growth depending on their activation state and the specific tumor context [4,10,11,12,14,15,21,22,24,25,26,27]. The role of TANs in cancer is complex, exhibiting both pro-tumor and anti-tumor characteristics, which are influenced by the tumor type, its stage, and specific signals from the TME [3,4,10,14,20,21,24,25,26,28]. TANs have a significant influence on angiogenesis, anti-tumor immunity, and the suppression of metastatic potential [4,10,11,12,14,15,16,22,23,24,25,26]. Meanwhile, specific subsets of neutrophils promote anti-tumor effects [4,11,14,15,21,22,24,25,28].

A key component of the TME is the desmoplastic reaction (DR), which is histologically characterized by the extensive proliferation of fibroblasts along with ECM components, primarily collagen [3,4,5,6,7,8,12,14,17,29]. A wide range of DRs is observed in breast carcinomas, varying from a cellular stroma rich in fibroblasts and myofibroblasts to a dense, acellular collagenous stroma [29,30]. This intricate procedure is driven by mechanisms involving paracrine activation of myofibroblasts, immune cytokine interactions, microvascular injury, and a bidirectional relationship [3,31]. Recent studies have highlighted the critical role of the DR in cancer progression, prognosis, and resistance to therapy [4,6,7,9,12,13,14,29]. The prognostic significance of cancer-associated fibroblasts (CAFs), key mediators of the DR, has also been emphasized in several tumor types, including breast carcinoma [6]. CAFs contribute to tumor growth, tissue infiltration, and metastatic potential by remodeling the ECM, secreting growth factors, and influencing the infiltration of immune cells [3,4,6,7,9,12,13,14,17,31].

Furthermore, emerging evidence indicates that distinct patterns of stromal reaction may markedly influence patient prognosis [6]. A well-studied case of this phenomenon was first observed in colorectal cancer, where specific DR types have been correlated with clinical outcomes [32]. In breast cancer, and more precisely in triple-negative breast cancer (TNBC), the presence of myxoid change and fibrotic focus within the tumor stroma has been shown to correlate with poorer overall and relapse-free survival. These stromal alterations not only serve as independent indicators of unfavorable prognosis but are also associated with reduced tumor-infiltrating lymphocyte density, highlighting a potential link between desmoplastic remodeling, immune evasion, and aggressive tumor behavior [33]. Similarly, the presence of myxoid stroma and hyalinized collagen at the invasive front of the tumor, which has been used to classify the DR into three categories (mature, intermediate, or immature) [33], has likewise been identified as an independent prognostic factor for recurrence-free survival [34,35]. Yet, the degree of stromal DR in breast cancer has been reported to correlate with both lymph node and distant organ metastasis, thereby significantly influencing patient prognosis [8,9,36]. Moreover, the tumor–stroma ratio has emerged as an independent prognostic parameter, with studies by Zakhartseva and Yanovytska [37] and Roeke et al. [38] demonstrating its strong association with overall, disease-free, and recurrence-free survival outcomes.

Previous research has extensively examined the individual roles of TANs and the DR in breast cancer, yet their potential interplay and combined influence on tumor biology and clinical outcomes remain poorly understood [4,14]. Despite growing evidence that both TANs and stromal remodeling significantly shape the TME and influence disease progression, the mechanistic and prognostic implications of their interaction have not been systematically investigated. In particular, the reciprocal relationship between TAN infiltration, the histological subtype of the DR, and established clinicopathological parameters, especially tumor grade, represents a promising yet largely unexplored field. Elucidating this interplay could provide novel insights into the biological underpinnings of tumor aggressiveness and identify potential prognostic or therapeutic biomarkers warranting further investigation.

To address this gap, the present study was designed as a rigorous, hypothesis-generating investigation to explore potential associations between these critical elements of the TME. TANs were quantified within specific stromal contexts (myxoid, keloid, and mature DR) in a cohort of breast cancer cases. The primary aim was to evaluate their correlation with tumor grade and other key clinicopathological features, providing preliminary data to support future larger-scale, multicenter validation studies. The novelty of this study lies in its detailed, histopathology-based characterization of both TANs and DR subtypes, and in examining their interaction and combined prognostic relevance in breast cancer. By focusing on a well-defined patient cohort and performing a meticulous stromal and immune cell analysis, this study seeks to generate compelling preliminary evidence and formulate new, testable hypotheses regarding the complex interplay within the TME, thereby laying the groundwork for subsequent large multicenter investigations.

## 2. Materials and Methods

### 2.1. Study Design

The study protocol was initially reviewed and approved by the Research Ethics Committee of the University of Western Macedonia (protocol number: 36/27 January 2023). Following approval, an invitation to participate was disseminated to the pathology unit of the public hospital G. Papanikolaou in the Prefecture of Thessaloniki, Greece. The invitation included a detailed description of the study’s objectives, significance, and methodology. Women who agreed to participate provided written informed consent, and all procedures were conducted in accordance with the ethical principles outlined in the Declaration of Helsinki (2013).

### 2.2. Participants’ Recruitment

Women with breast cancer were recruited from the pathology unit of the public hospital G. Papanikolaou in the Prefecture of Thessaloniki, Greece. Patients were eligible for inclusion if they had a histologically confirmed diagnosis of invasive breast carcinoma. Specimens were obtained as part of routine clinical care—core needle biopsies during the initial diagnostic workup and surgical resections at definitive management following confirmation of invasive breast carcinoma. Both core needle biopsy and surgical resection specimens were considered, with the invasive front of surgical specimens specifically evaluated to capture the most biologically active region of the TME, while the entirety of tumor tissue in core biopsies was assessed. Cases were required to have sufficient, well-preserved tissue to allow reliable histopathological evaluation, including assessment of stromal features and TANs. The exclusion criteria were as follows: the study excluded participants who had received preoperative (neoadjuvant) therapy, those diagnosed exclusively with ductal carcinoma in situ (DCIS) or lobular carcinoma in situ (LCIS) without an invasive component, and cases in which the available tissue material was insufficient or histological sections were of inadequate quality, thereby precluding reliable pathological evaluation. Additionally, cases of inflammatory breast carcinoma were also excluded, as this distinct subtype is characterized by a pronounced inflammatory TME that could potentially confound the evaluation of TANs.

### 2.3. Sample Size Calculation

The required sample size was estimated based on a similar study by Yanai et al. [33] in the breast cancer population. By using a one-way ANOVA test of significance (with the significance level at *p* < 0.05) to compare three DR groups and to achieve a power of 80%, we found that a total of 60 participants were required. To be more precise, for n = 60, the power was 0.865, assuming there was a 5% chance of making a type I error and a 13.5% chance of making a type II error, considering that the power was equal to 1-beta (1-beta ⇔ 1–0.865). Our study enrolled a total of 65 participants. Moreover, the group size allocation for the overall test was as follows: n = 24 for the immature DR, n = 16 for the intermediate DR, and n = 20 for the mature DR. In our study, we recruited and evaluated a total of 65 female patients with breast cancer who were assigned into 3 non-equal groups based on their desmoplasia classification (immature DR: n = 26, intermediate DR: n = 16, mature DR: n = 23).

### 2.4. Reduction in Potential Bias

To minimize potential sources of bias, several methodological precautions were implemented throughout the study. All histopathological evaluations were performed independently by two experienced pathologists who were blinded to the patients’ clinical and pathological data, ensuring objective and consistent assessment while eliminating observer bias. The same standardized criteria and magnification fields were applied consistently across all specimens to maintain analytical uniformity. Both biopsy and surgical resection samples were evaluated using predefined protocols, focusing on comparable stromal regions to minimize sampling variability. Data collection and statistical analyses were conducted independently, following a predefined analysis plan to prevent selective reporting. Additionally, all inclusion and exclusion criteria were clearly established prior to data analysis to avoid selection bias and ensure the integrity and reproducibility of findings.

### 2.5. Data Collection and Measurements

**Figure 1:**
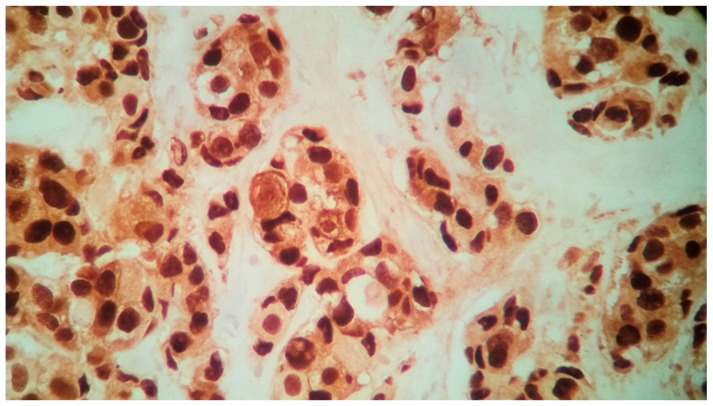
Representative immunohistochemical staining for ER in invasive NST breast carcinoma, demonstrating strong and diffuse nuclear positivity in >1% of neoplastic cells. Evaluation was performed according to ASCO/CAP guidelines. Original magnification ×400. Note: ER: Estrogen Receptor; NST: no special type.

**Figure 2:**
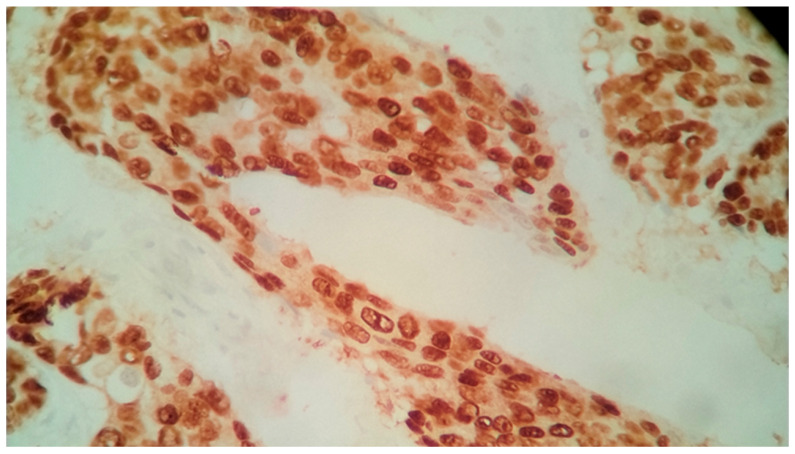
Representative immunohistochemical staining for PR in invasive NST breast carcinoma of no special type, showing intense and diffuse nuclear immunoreactivity in tumor cells. Scoring was performed according to ASCO/CAP guidelines, using a 1% cutoff for nuclear positivity. Original magnification ×400. Note: PR: progesterone receptor; NST: no special type.

**Figure 3:**
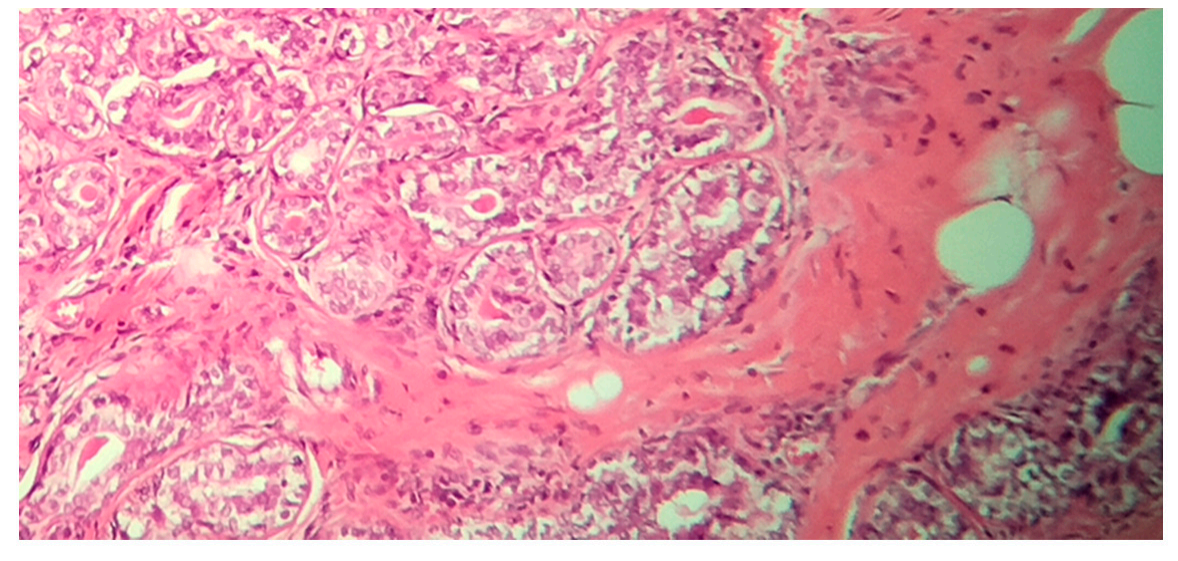
Representative histological image of invasive breast carcinoma, histological Grade 1, exhibiting a mature DR (H&Ε stain, original magnification ×400). The stromal component is composed of dense, well-organized eosinophilic collagen bundles and appears relatively hypocellular. In this case, TANs are sparse within the stromal compartment. Note: DR desmoplastic reaction; H&E: hematoxylin and eosin; TANs: tumor-associated neutrophils.

**Figure 4:**
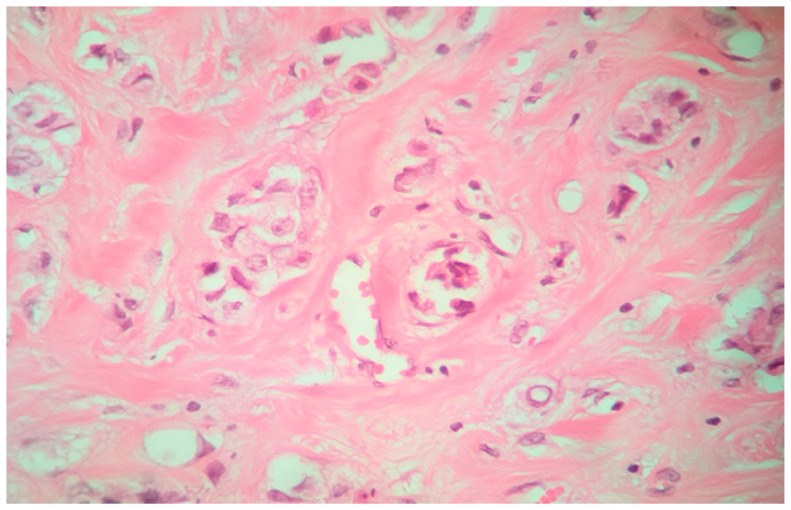
Representative histological image of invasive breast carcinoma, histological Grade 2, showing an intermediate (keloid-like) DR (H&E stain, original magnification ×400). The tumor stroma is characterized by thick, hyalinized, hypocellular eosinophilic collagen bundles. In this case, an intermediate level of TANs infiltration is observed within the stromal compartment. Note: DR desmoplastic reaction; H&E: hematoxylin and eosin; TANs: tumor-associated neutrophils.

**Figure 5:**
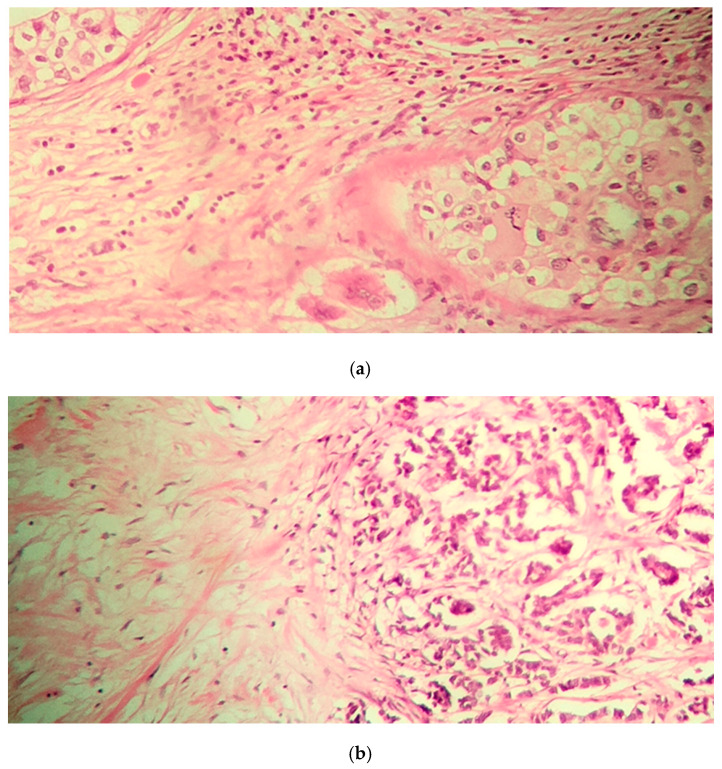
(**a**,**b**) Representative histological image of invasive breast carcinoma, histological Grade 3, demonstrating an immature (myxoid) DR (H&E stain, original magnification ×400). The stromal compartment shows an amorphous, basophilic extracellular matrix with absence of well-organized collagen fibers. In this case, numerous TANs are present within the myxoid stromal areas. Note: DR desmoplastic reaction; H&E: hematoxylin and eosin; TANs: tumor-associated neutrophils.

For each case, clinicopathological parameters were documented, including histological type according to the World Health Organization (WHO) classification [39,40], estrogen receptor (ER), progesterone receptor (PR), and cellular erythroblastic oncogene B2 (c-erbB2) status, as well as the presence of necrosis, perineural infiltration, and histological grade (Grade 1–3). Immunohistochemical evaluation of ER, PR, and human epidermal growth factor receptor 2 (HER2) was conducted on formalin-fixed, paraffin-embedded (FFPE) tissue sections as part of standard diagnostic procedures. The expression of ER and PR was assessed by immunohistochemistry and interpreted according to the guidelines of the American Society of Clinical Oncology and the College of American Pathologists (ASCO/CAP) [41]. Tumors were classified as hormone-receptor-positive if nuclear staining was present in 1% or more of the neoplastic cells. Examples of positive immunostaining for ER and PR are shown in Figure 1 and Figure 2. HER2 status was evaluated by immunohistochemistry and scored according to ASCO/CAP recommendations. Cases with an immunohistochemical score of 0 or 1+ were considered HER2-negative, whereas those with a score of 3+ were classified as HER2-positive. Tumors with an equivocal score (2+) were categorized as HER2-negative for this study, as confirmatory in situ hybridization (FISH) testing was not available. This conservative approach was adopted to avoid overestimation of HER2 positivity and to ensure consistent classification based solely on available data. Although the Ki-67 proliferation index was not available for all cases, histological grade according to the Nottingham grading system was used as the primary surrogate marker of tumor aggressiveness and proliferative activity. This system integrates tubule formation, nuclear pleomorphism, and mitotic count, and is widely regarded as a robust indicator of overall tumor biological behavior. TANs and the DR were evaluated on hematoxylin and eosin (H&E)-stained sections, 4–5 μm thick. TANs were quantified by counting neutrophils in ten consecutive high-power fields (×400) within areas of maximal infiltration (“hot spots”), while the DR was assessed at the invasive tumor front and classified as mature (Figure 3), intermediate (keloid-like) (Figure 4), or immature (myxoid) (Figure 5) based on established morphological criteria.

#### 2.5.1. Quantification of TANs

**Chart 1:**
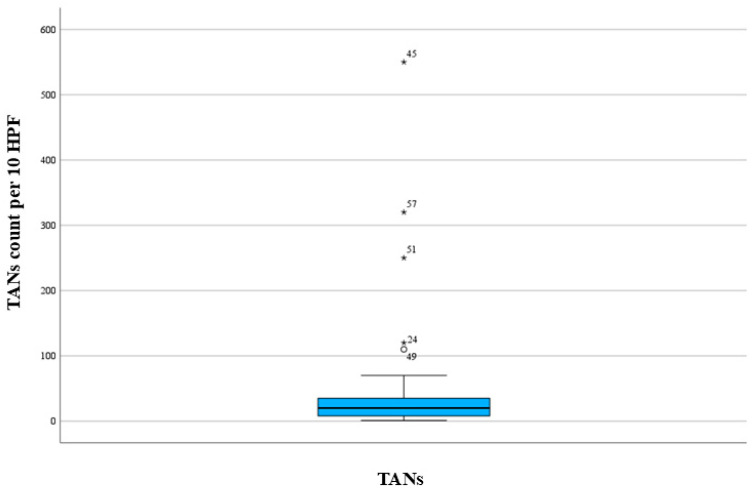
Sample distribution regarding TANs. Note: Each dot represents an individual measurement. Asterisks (*) denote outliers, defined as values lying more than 1.5× the interquartile range from the upper or lower quartile. TANs: tumor-associated neutrophils.

FFPE tissue blocks of invasive breast carcinoma were sectioned and stained with H&E. Tumor-associated inflammatory cells with polymorphonuclear morphology were identified based on established histomorphological criteria, including multilobed nuclei and granular cytoplasm. Accordingly, the counted cells are polymorphonuclear leukocytes (PMNs), as indicated by neutrophil morphology. For consistency with existing literature, these cells are referred to as TANs throughout the manuscript, acknowledging the inherent limitations of morphology-based identification. While immunohistochemical markers such as CD66b, myeloperoxidase (MPO), or CD15 can provide lineage-specific confirmation, the present study intentionally relied on H&E-based morphological assessment, reflecting routine diagnostic pathology practice and enabling broad applicability without additional technical requirements. This approach, although it does not allow discrimination among neutrophil subpopulations, has been widely used in exploratory and hypothesis-generating histopathological studies [42,43]. To minimize observer-related variability, all histopathological evaluations were independently performed by two experienced pathologists who were blinded to clinical and pathological data. Areas of maximal inflammatory infiltration (“hot spots”) were identified at low magnification, and PMNs/TANs were counted in ten non-overlapping high-power fields (×400 magnification). Cells located within vascular lumina, necrotic areas, or stromal regions without direct tumor contact were excluded from analysis. TAN counts ranged from 1 to 550 per 10 high-power fields, with a mean value of 40.35 (standard deviation 82.13) (Chart 1).

#### 2.5.2. Classification of DR

The DR was evaluated by histologically examining the connective tissue immediately adjacent to neoplastic cells, and cases were classified into three distinct categories: immature (myxoid stroma), intermediate (keloid-like stroma), and mature DR [30,33]. This three-tiered classification system has been extensively analyzed in colorectal cancer and subsequently adapted to breast carcinoma, where its prognostic relevance has also been reported [30,34,35]. The rationale for applying this system in the present study was based on fundamental biological similarities in CAF activation and ECM remodeling observed across various solid tumors, including breast cancer [8]. Notably, the presence of myxoid stromal change has been recognized as an adverse prognostic factor in triple-negative breast cancer [33,44]. To categorize cases within this system, the presence or absence of two key stromal characteristics was assessed at the invasive tumor front: (1) myxoid stroma, defined by an amorphous, mucinous, mildly basophilic or amphophilic ECM, and (2) keloid-like collagen, characterized by distinct, hypocellular, hyalinized eosinophilic bundles resembling those of keloid scars [30,33]. The absence of both features defined the mature type of DR, composed predominantly of delicate, multilayered collagen fibers [30]. This classification was chosen for its capacity to provide a detailed histological characterization of stromal maturity, thus offering a more precise framework for evaluating associations with TAN density compared to broader categorical approaches.

### 2.6. Statistical Analysis

All statistical analyses were conducted using IBM SPSS Statistics, version 25.0 (IBM Corp., Armonk, NY, USA). A two-tailed *p*-value of <0.05 was considered statistically significant. Descriptive statistics were employed to summarize both continuous and categorical variables. Continuous variables were presented as means, medians, standard deviations, interquartile ranges, and minimum and maximum values. Categorical variables were represented as counts (N) and percentages (%). The initial descriptive analysis assessed the distribution of TANs and the clinicopathological characteristics of the study population. To determine the appropriate statistical approach, tests for normality were performed. The Shapiro–Wilk test was applied to samples with sizes below 30, and the Kolmogorov–Smirnov test was used for those with sizes above 30. Because the distribution of TANs across most clinicopathological variables deviated significantly from normality (*p* < 0.05), non-parametric methods were employed for inferential analyses. For comparisons between two independent groups, the Mann–Whitney U test was used, as it does not require the assumption of a normal distribution. This test was applied to assess differences in TAN levels according to peri-neural invasion, necrosis, and hormone receptor status (ER and PR). For comparisons among three or more independent groups, the Kruskal–Wallis test was employed to evaluate differences in TAN distribution across categories such as DR type, histological grade, and c-erbB2 status. When statistically significant results were obtained, the Bonferroni post hoc test was performed to adjust for multiple comparisons. Associations between qualitative variables were analyzed using the Pearson chi-square (χ^2^) test, particularly to examine the relationship between DR type and histological grade.

## 3. Results

### 3.1. Clinicopathological Results

The clinicopathological data of the patients is summarized in Table 1. The majority of cases were classified as carcinomas of no specific type (NST), accounting for 86.2% (N = 56), while a smaller proportion consisted of invasive lobular carcinomas, at 13.8% (N = 9). In terms of tumor differentiation grade, 10.8% of the patients had Grade 1 tumors (N = 7), 41.5% had Grade 2 tumors (N = 27), and 47.7% had Grade 3 tumors (N = 31). Regarding hormone receptor status, 66.2% of the tumors were estrogen receptor-positive (ER+) (N = 43), and 50.8% were PR-positive (PR+) (N = 33). For c-erbB2 status, 46.2% of the tumors were reported as negative (1+) (N = 30), 18.5% as weakly positive (2+) (N = 12), and 35.4% as positive (3+) (N = 23). Necrosis was identified in 29.2% of the tumors (N = 19), and perineural invasion was observed in 35.4% (N = 23). The study of DR revealed that the most common finding was myxoid stroma, observed in 40% of cases (N = 26). This was followed by mature DR, noted in 35.4% of cases (N = 23), and the keloid-like (hyalinized) stroma was less frequently seen in 24.6% of cases (N = 16).

### 3.2. Association Between TANs, DR, and Tumor Grade

**Chart 2:**
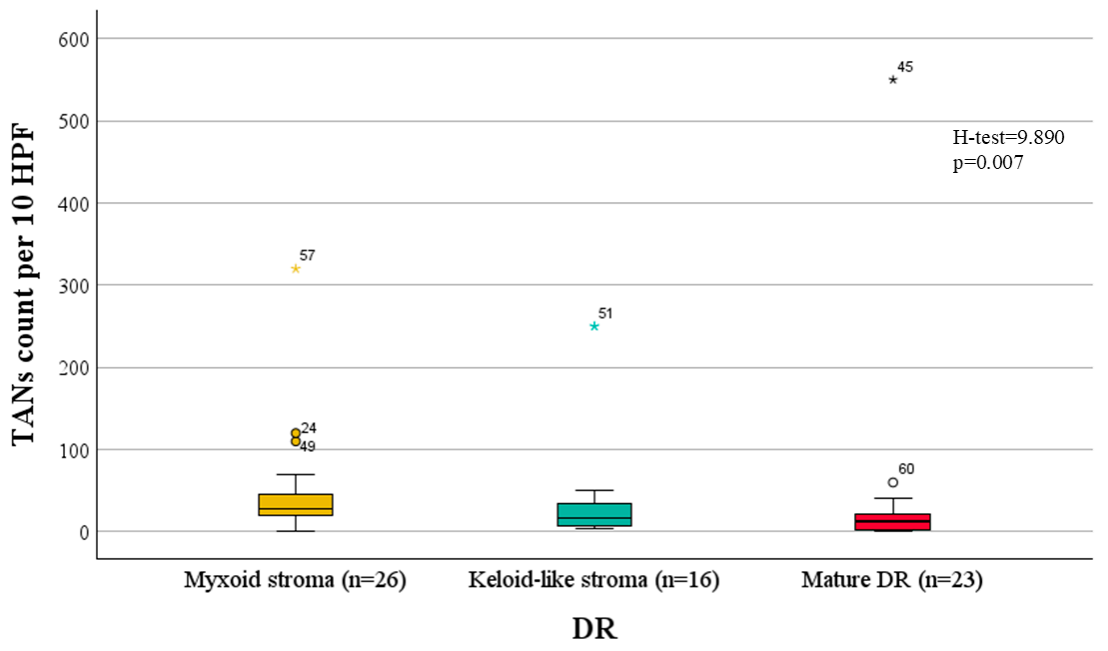
Distribution of TANs according to DR type. Note: Each dot represents an individual measurement. Asterisks (*) denote outliers, defined as values lying more than 1.5× the interquartile range from the upper or lower quartile. TANs: tumor-associated neutrophils; DR: Desmoplastic reaction.

A significant association was observed between TAN levels and the type of DR surrounding the tumor (Kruskal–Wallis H = 9.890, *p* = 0.007) (Chart 2). Post hoc analysis demonstrated that tumors with myxoid stroma exhibited significantly higher TAN infiltration compared with those displaying a mature stroma (mean rank = 41.58 vs. 24.80, *p* = 0.006) (Table 2).

**Chart 3:**
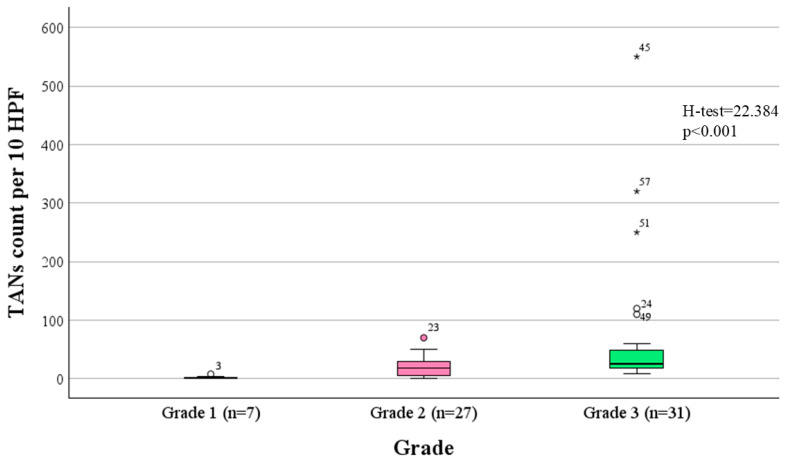
Distribution of TANs according to histological grade. Note: Each dot represents an individual measurement. Asterisks (*) denote outliers, defined as values lying more than 1.5× the interquartile range from the upper or lower quartile. TANs: tumor-associated neutrophils.

A strong stepwise relationship was also identified between TAN levels and tumor grade (Kruskal–Wallis H = 22.384, *p* < 0.001). TAN counts increased progressively with tumor grade: Grade 2 tumors showed significantly higher TAN levels than Grade 1 tumors (mean rank = 29.24 vs. 6.43, *p* = 0.013), while Grade 3 tumors exhibited the highest TAN infiltration overall, significantly exceeding both Grade 1 (*p* < 0.001) and Grade 2 tumors (*p* = 0.026) (Table 3, Chart 3).

### 3.3. Association Between TANs and c-erbB2 Status

**Chart 4:**
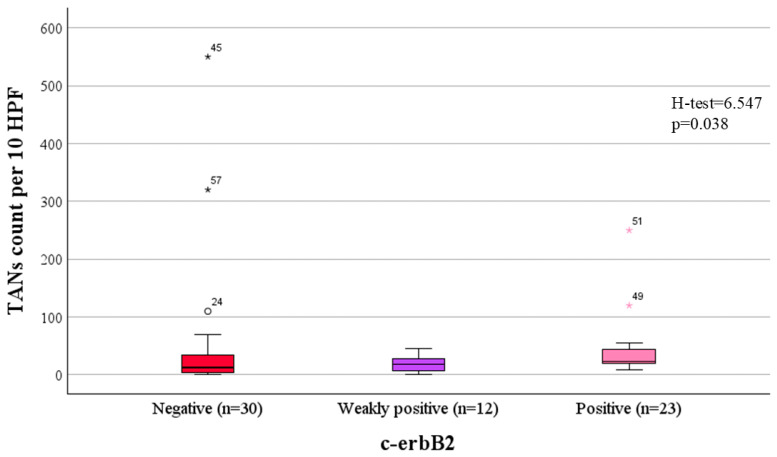
TANs distribution based on c-erbB2. Bars represent the median TAN count for each group. Note: Each dot represents an individual measurement. Asterisks (*) denote outliers, defined as values lying more than 1.5× the interquartile range from the upper or lower quartile. c-erbB2: cellular erythroblastic oncogene B2; TANs: tumor-associated neutrophils.

Analysis of TAN levels in relation to c-erbB2 status revealed significant differences among the various HER2 expression categories (Kruskal–Wallis H = 6.551, *p* = 0.038). Although post hoc comparisons did not reach statistical significance after Bonferroni correction, a clear trend toward higher TAN counts was observed in the c-erbB2-positive group compared with the c-erbB2-negative group (mean rank = 36.65 vs. 28.91, *p* = 0.059) (Table 4, Chart 4). While the current study lacks sufficient statistical power to confirm a definitive association, this observed tendency suggests a potentially meaningful biological relationship between TAN infiltration and c-erbB2 positivity that warrants further investigation in larger, multicenter cohorts.

### 3.4. Association Between TANs and Other Clinicopathological Features

**Chart 5:**
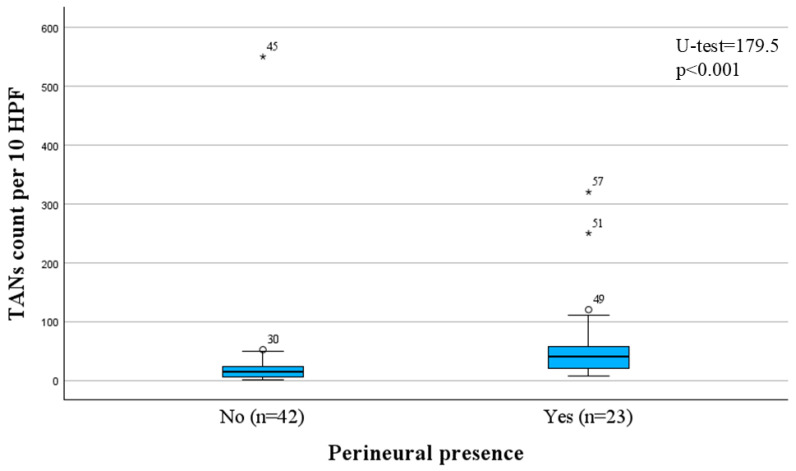
Distribution of TANs based on perineural invasion. Note: Each dot represents an individual measurement. Asterisks (*) denote outliers, defined as values lying more than 1.5× the interquartile range from the upper or lower quartile. TANs: Tumor-Associated Neutrophils.

**Chart 6:**
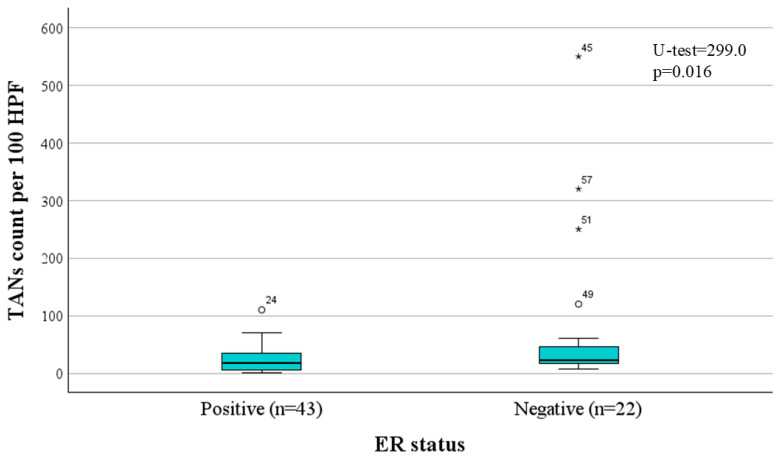
Distribution of TANs based on ER. Note: Each dot represents an individual measurement. Asterisks (*) denote outliers, defined as values lying more than 1.5× the interquartile range from the upper or lower quartile. TANs: Tumor-Associated Neutrophils; ER: estrogen.

**Chart 7:**
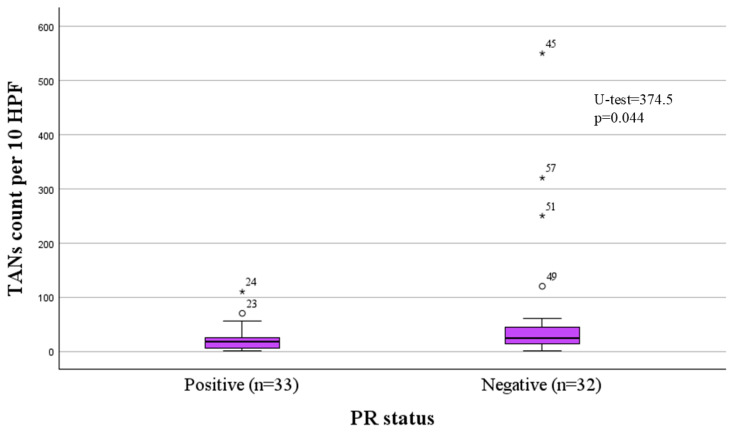
Distribution of TANs based on PR. Note: Each dot represents an individual measurement. Asterisks (*) denote outliers, defined as values lying more than 1.5× the interquartile range from the upper or lower quartile. TANs: Tumor-Associated Neutrophils; PR: progesterone.

**Chart 8:**
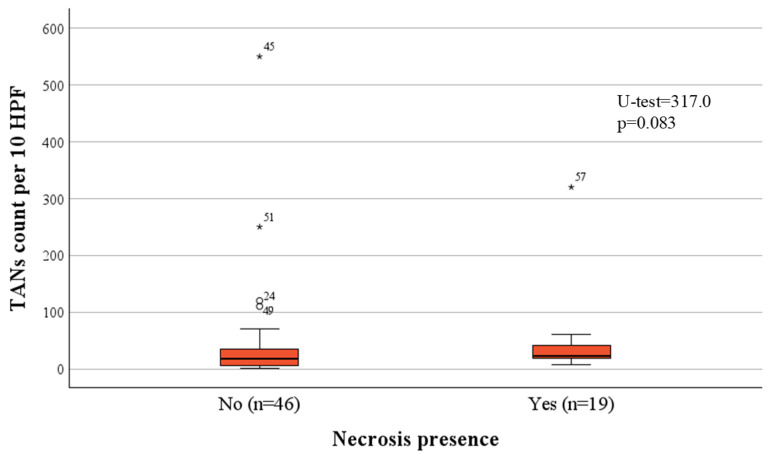
Distribution of TANs based on necrosis. Note: Each dot represents an individual measurement. Asterisks (*) denote outliers, defined as values lying more than 1.5× the interquartile range from the upper or lower quartile. TANs: Tumor-Associated Neutrophils.

TAN levels showed several statistically significant associations with key clinicopathological parameters (Table 5, Chart 5, Chart 6, Chart 7 and Chart 8). Tumors with perineural invasion exhibited significantly higher TAN counts compared to those without this feature (Mann–Whitney U = 179.5, *p* < 0.001). Additionally, ER-negative (Mann–Whitney U = 299, *p* = 0.016) and PR-negative (Mann–Whitney U = 374.5, *p* = 0.044) tumors showed significantly increased TAN infiltration compared to hormone-receptor-positive tumors. In contrast, the presence of tumor necrosis did not show a significant correlation with TAN levels (*p* = 0.083). In summary, these findings suggest that increased TAN infiltration is associated with more aggressive pathological features, particularly perineural invasion and hormone receptor negativity.

### 3.5. Association Between DR and Tumor Differentiation Grade

**Chart 9:**
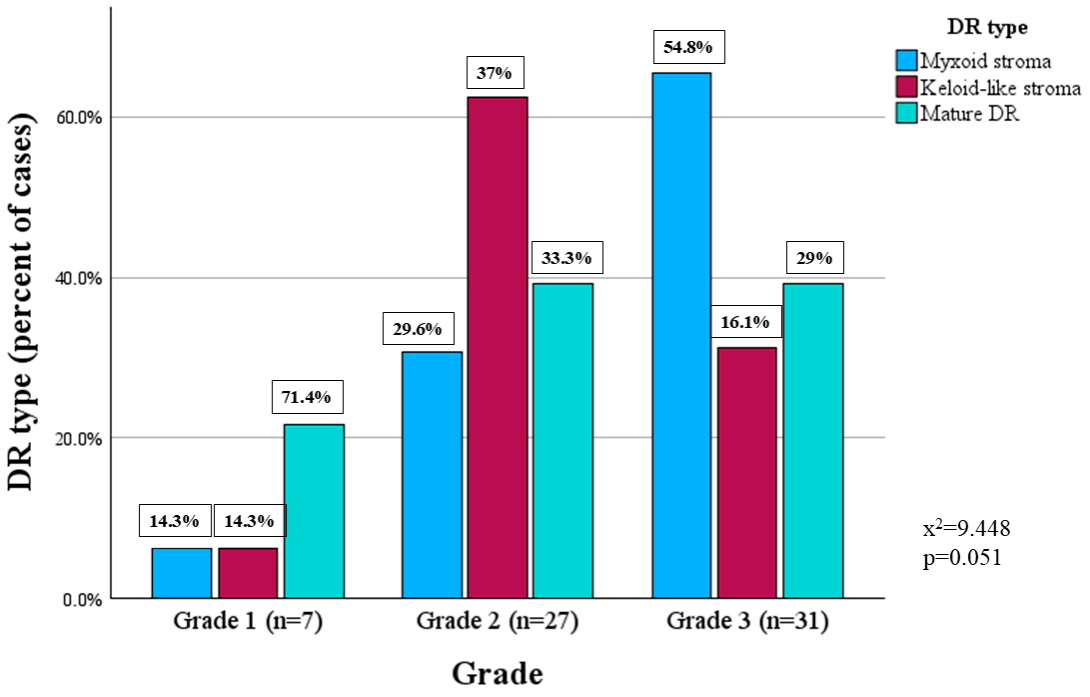
Frequency distribution of DR desmoplastic reaction based on the degree of differentiation (Grade).

A statistically significant correlation was observed between DR type and tumor differentiation grade (Pearson χ^2^ = 9.448, *p* = 0.051) (Table 6, Chart 9). Grade 1 tumors were most frequently associated with a mature DR pattern (71.4%), whereas Grade 2 tumors more commonly exhibited a keloid-like stroma (37%). In contrast, Grade 3 tumors predominantly demonstrated a myxoid stromal pattern (54.8%). Although this association did not reach the conventional threshold for statistical significance, the observed distribution suggests a potential relationship between increasing tumor grade and progressive stromal immaturity.

## 4. Discussion

Recent scientific attention has shifted from focusing solely on cancer cells to examining the various cellular and non-cellular components of the TME. This change emphasizes the active role the TME plays in the cancer process, influencing tumor formation, metastatic potential, and the effectiveness of treatment therapies [4,5,6,7,9,12,13,14,17,29,45]. Within this context, the present hypothesis-generating study aimed to investigate the relationship between TANs, distinct patterns of DR, tumor grade, and other key clinicopathological parameters routinely assessed in breast cancer. Although preliminary in nature, the findings from this cohort of invasive breast carcinomas provide valuable insight into the complex cellular interactions within the breast cancer TME. Specifically, the observed associations between certain DR subtypes, elevated TAN infiltration, and more aggressive pathological features suggest a potential biological interplay that warrants validation in larger, multicenter investigations.

The heterogeneity of the TME across different cancer types is well established, with each malignancy exhibiting distinct tumor-specific characteristics [3,4,6,7,12,14]. Extensive research has explored the TME in malignancies such as melanoma, lung, and colorectal carcinomas, where a pronounced inflammatory response is frequently observed [23]. Beyond these malignancies, breast cancer, although generally characterized by a milder inflammatory response, underscores the pivotal influence of the TME in driving carcinogenesis and determining tumor biological behavior [5,14,23]. Notably, the importance of tumor-infiltrating lymphocytes (TILs) in breast cancer is now well understood and has been formally included in the 5th edition of the WHO Classification of Breast Tumors in 2019 [40]. The present study contributes to this evolving field by elucidating the complex interplay between TANs and DR within the breast cancer TME. The observed associations between these stromal elements, tumor grade, and other key clinicopathological features highlight their potential prognostic significance and suggest new avenues for targeted therapeutic intervention [3,4,9,11].

The results of our preliminary study confirm the heterogeneity of TAN infiltration [10,14], as we observed a non-normal distribution of TAN counts across various clinicopathological categories. There were statistically significant differences in TAN levels among the three types of DR, with significantly higher TAN counts detected in myxoid stroma compared to the mature DR. This finding supports the growing evidence that the specific composition and stiffness of the ECM play a critical role in the recruitment and polarization of immune cells within the TME [3,4,5,6,7,8,11,12,13,17,46]. In this context, the increased density of TANs observed in myxoid immature stroma supports a pro-tumorigenic role for TANs in less differentiated, biologically aggressive TMEs [6,7,12,14,17,44]. Indeed, aggressive tumor phenotype has been frequently associated with myxoid immature DR [8,33,44], which is characterized by a loose, amorphous, mucin-rich ECM that may create a permissive niche for neutrophil infiltration through altered chemokine gradients and reduced physical constraints [6,7,10]. The association between the myxoid stromal subtype and increased TAN infiltration provides further insight into neutrophil adaptive behavior within the TME. The biochemical composition and structural organization of the ECM are key determinants of immune cell recruitment and functional polarization. In particular, the highly hydrated and structurally permissive nature of immature myxoid stroma may favor the accumulation of TANs and support their polarization toward a pro-tumorigenic (N2-like) phenotype. In contrast to anti-tumor (N1) neutrophils, these N2-like TANs are thought to undergo functional reprogramming in response to stromal-derived signals, including transforming growth factor-β (TGF-β) [47,48]. Although neutrophil phenotypes were not directly assessed in the present study, our findings are consistent with the concept that stromal architecture and ECM characteristics critically shape immune cell behavior within aggressive breast cancer TMEs. Conversely, TANs in this context are unlikely to remain static; instead, they may change the characteristics of the stroma [8,20,33]. This alteration is achieved through the secretion of cytokines, chemokines, or enzymatic activity, which modifies the ECM. As a result, we can observe a feedback loop that promotes the growth of cancer [4,6,7,10,12,13,14,17,49]. The fact that we see high neutrophil density exactly where the stroma is most immature strongly suggests that aggressive tumors are actively reshaping their local environment to pave the way for metastasis. Furthermore, when considering this concept more broadly, the mature DR, characterized by well-organized collagen fibers, may contribute to the formation of a more stable environment or even an anti-tumor layer, which is associated with reduced TAN infiltration [8,49].

Furthermore, our study found a statistically significant association between TAN counts and tumor grade. We prioritized histological grade over molecular subtyping based on Ki-67, as histological grade integrates multiple architectural and cytological parameters, including tubule formation, nuclear pleomorphism, and mitotic activity, thereby providing a comprehensive assessment of tumor differentiation and biological aggressiveness. In contrast, Ki-67 evaluation is known to be subject to considerable inter-observer and inter-laboratory variability [50], particularly in retrospective settings, as well as by methodological inconsistencies, the absence of universally accepted scoring standards, and heterogeneous incorporation into multigene panels [51]. Accordingly, the use of histological grade enabled a multifaceted, standardized evaluation of tumor biology that was consistently applicable across the entire study cohort. Grade 3 tumors had the highest TAN counts compared to both Grade 1 and Grade 2 tumors. The strong association between high TAN density and histological Grade 3 tumors (*p* < 0.001) supports the concept that TAN infiltration is associated with aggressive tumor phenotypes. High-grade breast carcinomas are known to exhibit an inflammatory TME enriched in CXC-family chemokines, such as CXCL8 (IL-8) [52] and CXCL1 [53], which may promote neutrophil recruitment independently of hormone receptor status. This may explain why ER negativity, although commonly associated with higher histological grade [54], did not show a strictly concordant pattern with TAN density or DR in our cohort. Importantly, ER positivity in breast cancer, while generally associated with a more favorable prognosis, demonstrates substantial biological heterogeneity, and its prognostic significance is strongly modified by histological grade and tumor stage [54]. ER-positive/PR-negative and low-ER tumors are often more aggressive, exhibiting higher histological grades, HER2 positivity, and advanced disease stages, with characteristics similar to ER-negative carcinomas [54,55,56]. This heterogeneity in ER expression provides a plausible explanation for the observed association between ER positivity and Grade 3 tumors in our cohort and underscores that hormone receptor status, immune infiltration, and stromal remodeling represent overlapping yet biologically distinct dimensions of breast cancer heterogeneity [54]. Further, TAN infiltration and stromal remodeling illustrate the dynamic interactions between the immune system and the stroma, which are not solely determined by tumor cell differentiation or hormone receptor expression. Although the literature describes TANs in aggressive tumors as often having a predominantly N2-like functional profile, characterized by the secretion of matrix-remodeling enzymes like matrix metalloproteinase 9 (MMP-9) and pro-angiogenic factors such as vascular endothelial growth factor (VEGF), which facilitate the remodeling of the ECM and promote angiogenesis, these functional characteristics were not directly assessed in the current study. They are mentioned here solely to provide biological context for the observed associations [3,4,10,14,20,21,24,25,26,28].

Additionally, Grade 2 tumors exhibited higher TAN levels than Grade 1 tumors. The Grade 1 tumor group was the smallest in our cohort, consisting of only 7 patients. This group was found to display a mature DR more frequently (71.4%) and was also associated with the lowest TAN counts. This finding is consistent with existing knowledge that more intense TAN infiltration is linked to more aggressive tumors and worse prognosis in breast cancer and other types of malignancies [4,5,10,11,12,14,15,16,20,21,22,24,25,26,27,28,46]. However, it is important to acknowledge that the small size of the Grade 1 subgroup in our cohort necessitates confirmation in larger cohorts to ensure the robustness of these results.

Regarding the hormone status of the tumors, our study showed that statistically significantly higher TANs levels infiltrated the ER-negative and PR-negative tumors. These findings align with the clinical characteristics of these tumors, as ER-negative and PR-negative tumors (particularly triple-negative breast cancer) tend to exhibit more aggressive biological behavior, leading to a poorer prognosis for patients [14,15,16,46,57,58,59,60]. The increased number of TANs in these aggressive tumor types supports the idea that TANs contribute to a more hostile and pro-tumorigenic microenvironment [4,14,16,20,24,25,46,57,58,59,60]. In contrast, no statistically significant association was observed between hormone receptor status and either TAN infiltration or DR patterns. This finding suggests that neutrophil recruitment and stromal remodeling may be regulated by biological pathways that are at least partly independent of hormonal signaling, indicating that these components of the TME represent distinct and autonomous dimensions of tumor biology. Such dissociation underscores the complexity of tumor–stroma–immune interactions and supports the notion that stromal and immunological alterations can emerge across diverse molecular backgrounds [61,62]. Although these observations constitute preliminary evidence and require validation in larger cohorts, they provide a compelling basis for generating new, testable hypotheses regarding TME dynamics [61]. Importantly, the potential independence of TAN infiltration and DR from hormone receptor status may have broader clinical relevance. The identification of aggressive stromal patterns across different breast cancer subtypes suggests that stroma-oriented therapeutic or surveillance strategies could be considered for high-risk patients based on routine histopathological assessment of H&E-stained sections, without reliance on costly molecular assays [30,63]. Furthermore, these TME features may serve as practical markers for patient stratification and closer clinical monitoring, irrespective of molecular subtype [64].

Besides tumor grade, we examined potential associations between TAN levels and various clinicopathological features commonly assessed in clinical practice. Regarding c-erbB2 status, our findings suggest that c-erbB2-positive tumors tend to have higher counts of TANs. This observation is particularly noteworthy, as c-erbB2-positive tumors are typically more aggressive and exhibit distinct molecular characteristics [15,16,58]. Further investigation of this relationship is warranted.

Yet, our study showed markedly increased levels of TANs in higher-grade (Grade 2 and 3) and hormone-receptor-negative tumors, underscoring their pivotal role in promoting breast cancer progression [20,58]. This observation aligns with the established pro-tumorigenic functions of TANs, including the stimulation of angiogenesis, ECM remodeling through the secretion of MMPs, and facilitation of immune evasion [3,4,5,6,7,8,10,12,13,14,15,17,22,24,25,46,65]. These mechanisms may account for the observed association between elevated TAN counts and more aggressive, high-grade tumor phenotypes. Moreover, the notable trend of higher TAN counts in c-erbB2-positive tumors may suggest a synergistic effect between oncogenic signaling and neutrophil-mediated pro-tumorigenic functions [3,4,7,58,59]. Although the association between TANs and c-erbB2 positivity did not achieve statistical significance, it remains an intriguing trend that warrants further investigation in larger multicentric studies. This is especially true considering that our cohort size may have limited the study’s power. Additionally, it is worth noting that this trend did not reach statistical significance after post hoc correction in our study. This lack of significance might be attributed to the conservative nature of the Bonferroni correction method or the small sample size in the subgroups. Further research involving larger breast cancer populations is needed to explore this trend.

The presence of perineural invasion was also strongly associated with significantly higher TAN counts. Perineural invasion is recognized as a poor prognostic indicator, as it serves as a primary route for metastasis [3,4,14,21,65,66]. Our preliminary findings suggest a strong correlation between perineural invasion and increased TAN counts, indicating that TANs may play a critical role in the metastatic process [4,14,20,21,22,23,24,25,26,28,66]. TANs may infiltrate nerves by secreting enzymes, such as matrix metalloproteinases, which promote the degradation of the perineural sheath. This degradation can create pathways for cancer cells to follow along the nerves. Still, TANs can secrete pro-migratory factors that enhance the invasive capabilities of tumor cells within the perineural niche [65,66]. Although no significant association was identified between TAN levels and the presence of necrosis, this finding may, at least in part, reflect methodological considerations. Specifically, as noted earlier, neutrophils located within necrotic debris were deliberately excluded from the counts to minimize potential overestimation. Consequently, this approach may have limited the detection of associations between TAN density and necrotic or hypoxic tumor regions. Future studies employing immunohistochemical (IHC) techniques could provide more accurate delineation of the spatial relationship between neutrophil infiltration, hypoxia, and necrosis, thereby offering more profound insights into the interplay between inflammatory and metabolic components of the TME.

Our study also found an association that approaches statistical significance between the type of DR and the tumor grade. Based on previous studies that have categorized stromal types in breast cancer [44], we observed a clear gradient in DR, ranging from mature stroma to myxoid, as tumor grade increases. This significant finding supports the notion that the evolving characteristics of the stroma are closely related to tumor aggressiveness [8,30,33,44].

Our results indicate that mature stroma is more commonly found in Grade 1 tumors, whereas Grade 2 tumors typically exhibit hyalinized (keloid-like) stroma, and Grade 3 tumors are characterized by myxoid stroma [44]. Mature stroma, which features fine, multilayered collagen fibers, may create a more organized environment that potentially limits tumor growth [30]. In contrast, the myxoid stroma, composed of an amorphous, mucin-rich ECM, likely signifies an immature and less organized stroma that can facilitate tumor cell infiltration and increase the potential for metastasis [5,7,8,13,17,27,29,33,44]. Keloid-like stroma, often observed in Grade 2 tumors, may represent a shift toward a more mature and pro-tumorigenic microenvironment [44,67]. This aligns with previous studies that have linked sclerotic stromal patterns to adverse prognostic features in breast cancer [44,67]. Consequently, the varying types of desmoplastic stroma observed across different tumor grades underscore the importance of a thorough evaluation of the TME for accurate prognostic stratification [9].

The findings discussed have important potential clinical implications. We observed significant associations between higher TAN counts and aggressive tumor characteristics, including hormone receptor negativity, c-erbB2 positivity, and perineural invasion. Taking together, these observations suggest that TME features, such as TAN density and DR patterns, may reflect underlying tumor aggressiveness at the time of diagnosis. On this basis, we propose the concept of a “TAN–DR profile,” combining TAN density with stromal subtype, as a morphology-based framework for capturing TME heterogeneity. While this profile cannot yet be considered a validated prognostic tool, it may represent a cost-effective and widely accessible approach for exploratory risk stratification using routine H&E-stained sections. It could also identify patients with more aggressive underlying tumor biology who might benefit from intensified treatment options [34].

A semi-quantitative score could be the basis of such a prognostic model. For instance, it could be based on the creation of a composite score from 0 to 2, combining the scoring for high TAN counts (e.g., >5 TAN/HPF = 1 point) and for an immature DR (myxoid layer = 1 point). In this model, patients with a “high-risk” profile (e.g., a score of 2, indicating an abundance of TANs within a myxoid stroma) are predicted to have a more aggressive disease and a poorer prognosis. We note that a rigorous statistical analysis, such as ROC curve analysis in a large validation cohort, should be employed to develop and validate specific cutoffs, thereby transforming this observation from a promising association to a validated clinical tool [68,69].

The creation and validation of this hypothetical profile would require a phased approach:

✓ Phase 1: The next immediate step is to validate our findings in a larger, multicenter cohort of breast cancer patients. The observed associations need to be confirmed to allow for refinement of a scoring system.
✓ Phase 2: For clinical applicability, subjective assessment must be minimal. In this phase, we would include the development and validation of a standardized protocol for IHC for neutrophil markers (e.g., CD66b, MPO) and digital pathology algorithms to automate the quantification of TANs and the classification of stromal types [68].
✓ Phase 3: The most definitive validation would come from a prospective clinical trial. This trial would not only validate the TAN–DR profile but also assess its potential prognostic and predictive power [70]. Specifically, it would evaluate whether the TAN–DR profile can predict patient outcomes independently of other prognostic markers.

These steps could help convert the TAN–DR profile from a promising observation into a validated and clinically useful biomarker that influences treatment decisions in breast cancer.

To sum up, this study has both strengths and limitations. Firstly, a key strength is that this study presents an integrative analysis of TAN density in conjunction with three histologic DR subtypes, correlating these with tumor grade and main clinicopathological variables (ER/PR, c-erbB2, and perineural invasion), thereby addressing an underexplored area in breast cancer research. Secondly, the methodology includes standardized techniques for hot-spot TAN quantification across ten high-power fields and a clearly defined three-tier DR classification based on conventional H&E sections, enhancing reproducibility. Thirdly, internal validity is strengthened by blinded histopathological assessments, explicit inclusion and exclusion criteria, and the use of appropriate non-parametric statistics with the Bonferroni correction to minimize bias and support robust findings.

Considering the study’s limitations, firstly, this was a single-center investigation; therefore, the generalizability of our findings to broader breast cancer populations remains limited and requires validation in multicenter studies including more diverse patient cohorts. Secondly, although the overall sample size was sufficient to identify the primary associations examined, the unequal distribution of cases across DR subgroups may have influenced the sensitivity of our pairwise comparisons. Specifically, smaller subgroup sizes, such as those in the intermediate (keloid-like) category, may have limited our statistical power to detect subtle differences. While the application of Bonferroni correction minimized the risk of Type I errors and ensured the robustness of significant findings, this conservative approach may have increased the likelihood of Type II errors, underscoring the need for larger studies with more balanced subgroup sizes to validate these preliminary observations. Thirdly, another limitation is the study’s retrospective design, which carries an inherent risk of selection bias and limits the ability to control for potential confounding factors fully. Consequently, prospective studies are required to confirm the observed associations and to clarify their prognostic significance.

Fourthly, Ki-67 proliferation index data were not uniformly available for all cases, as Ki-67 assessment was not routinely performed at the time of diagnosis for a subset of patients included in this retrospective cohort. To avoid bias from incomplete data, Ki-67 was not included in the analyses, and histological grade was used as a surrogate marker of tumor aggressiveness. In addition, functional assays aimed at elucidating the underlying biological mechanisms were not performed. Fifthly, the study population consisted exclusively of NST and invasive lobular carcinomas. However, these represent the most common histological subtypes of breast cancer; the applicability of our findings to rarer histological variants may be limited. Lastly, HER2 status was assessed via immunohistochemistry, and equivocal (2+) cases were conservatively classified as negative due to the absence of confirmatory FISH testing. This methodology may have reduced the capacity to identify associations involving TANs.

Despite these limitations, we present our results as a robust starting point for further investigation. The significant associations identified, particularly between tumor-associated neutrophils and tumor grade (*p* < 0.001), provide strong foundational evidence for larger, multicenter, prospective studies to validate and extend these findings.

## 5. Conclusions

The findings of our exploratory study highlight significant histological associations between TANs, types of DR, tumor grade, and various clinicopathological features in breast cancer. We found that aggressive tumor phenotypes, such as higher-grade tumors, c-erbB2 positivity, hormone receptor negativity, and perineural invasion, along with specific stromal patterns, like myxoid stroma, are consistently associated with higher counts of TANs. This suggests that TANs play a critical role in breast cancer progression and the dynamic remodeling of the TME [3,4,7,8,10,13,14,15,20,22,25,71].

Given that this study provides a cross-sectional, pathology-based analysis, our findings should be viewed as a morphological “snapshot” of tumor–stroma–immune interactions at the time of diagnosis rather than as evidence of causal or prognostic effects. Although we cannot predict long-term clinical outcomes, the consistent presence of high TAN density and immature stromal architecture, alongside established markers of aggressiveness, suggests that these features of the TME reflect the tumor’s current biological state and its potential for progression. While the “TAN–DR profile” cannot yet be considered a validated prognostic tool, our results indicate it may serve as a biologically significant indicator of tumor aggressiveness at presentation and merit further investigation.

Further studies are required to elucidate the molecular mechanisms underlying TAN plasticity across different stromal contexts [25]. Improved mechanistic insight may ultimately contribute to the development of novel biomarkers and therapeutic strategies targeting key TME components [3,4,7,10,12,13,14,15,25,71].

On this basis, the dynamic interplay between TANs and DR may inform the rational design of novel, targeted therapeutic strategies, a proposition that warrants elucidation and validation in future mechanistic and prospective clinical studies [4,6,7,9,10,12,14,15,22,24,25,26,27,28,45]. For instance, we could target neutrophil recruitment or function if TANs are identified as pro-tumorigenic in the myxoid stroma of breast cancer cases with this specific stromal subtype [4,5,7,8,14,21,24,25]. Promising therapeutic options could also include blocking chemokine receptors on neutrophils, inhibiting cytokines that activate neutrophils, and depleting specific tumor-inducing subsets of TANs [3,4,7,10,11,14,15,21,24,25,26,27]. Likewise, understanding how TANs enhance the remodeling of the ECM in higher-grade tumors could lead to the development of effective therapies. These therapies might target enzymes involved in collagen degradation and cross-linking, which make the ECM stiffer and inhibit cancer cell infiltration [4,5,6,7,11,12,13,14,17,71]. Still, combining the inhibition of tumor cell invasion with the blockade of TAN-mediated support could be explored as a potential treatment strategy for tumors that exhibit perineural invasion [72]. These relationships present the potential to develop personalized therapeutic approaches tailored to the specific characteristics of the TME in individual breast cancer patients [5]. In conclusion, our hypothesis-driven study provides robust foundational observations that require validation in larger, multicenter, prospective cohorts to determine their clinical relevance and potential prognostic value.

## Figures and Tables

**Table 1:** Clinicopathological characteristics for the entire sample, N = 65.

		N	%
Histological type	NST	56	86.2
	Lobular	9	13.8
Necrosis	No	46	70.8
	Yes	19	29.2
Perineural invasion	No	42	64.6
	Yes	23	35.4
DR	Immature DR (presence of myxoid stroma)	26	40.0
	Intermediate DR (presence of keloidal stroma)	16	24.6
	Mature DR	23	35.4
ER	Positive	43	66.2
	Negative	22	33.8
PR	Positive	33	50.8
	Negative	32	49.2
c-erbB2	Negative	30	46.2
	Weakly positive	12	18.5
	Positive	23	35.4
Degree of differentiation (Grade)	Grade 1	7	10.8
	Grade 2	27	41.5
	Grade 3	31	47.7

Note: NST: no specific type; DR: Desmoplastic reaction; ER: estrogen; PR: progesterone; c-erbB2: cellular erythroblastic oncogene B2. Data are expressed as number of cases (percentage).

**Table 2:** Results of the Kruskal–Wallis test for evaluating the association of TANs with DR type.

							Post Hoc Comparisons		
	DR	N	Median	Mean Rank	Kruskal–Wallis H	*p*-Value	Myxoid Stroma vs. Mature DR	Keloid-like Stroma vs. Mature DR	Myxoid Stroma vs. Keloid-like Stroma
**TANs**	Myxoid stroma	26	28.5	41.58	9.890	0.007 *			
	Keloid-like stroma	16	16.5	30.84			0.221		
	Mature DR	23	12	24.80			0.006 *	0.978	

Note: ΤAΝs: Tumor-Associated Neutrophils; DR: Desmoplastic reaction. * *p* < 0.05 indicates statistical significance.

**Table 3:** Results of the Kruskal–Wallis test to evaluate the correlation of TANs with the degree of differentiation grade.

							Post Hoc Comparisons		
	Degree of Differentiation (Grade)	N	Median	Mean Rank	Kruskal–Wallis H	*p*-Value	1	2	3
**TANs**	Grade 1	7	1	6.43	22.384	<0.001 *			
	Grade 2	27	19	29.24			0.013 *		
	Grade 3	31	25	42.27			<0.001 *	0.026 *	

Note: ΤAΝs: Tumor-Associated Neutrophils. * *p* < 0.05 indicates statistical significance.

**Table 4:** Results of the Kruskal–Wallis test for evaluating the association of TANs with c-erbB2.

							Post Hoc Comparisons		
	c-erbB2	N	Median	Mean Rank	Kruskal–Wallis H	*p*-Value	Negative	Weakly Positive	Positive
**TANs**	Negative	30	12	28.87	6.547	0.038 *			
	Weakly positive	12	19	27.83			1.000		
	Positive	23	23	41.09			0.059 *	0.147	

Note: * *p* < 0.05 indicates statistical significance. ΤAΝs: Tumor-Associated Neutrophils; c-erbB2: cellular erythroblastic oncogene B2.

**Table 5:** Results of the Mann–Whitney test for evaluating differences in TANs in relation to perineural invasion, necrosis, ER and PR.

		N	Median	Mean Rank	Mann–Whitney U	*p*-Value
**TANs**	**Perineural invasion**					
	*No*	42	15	25.77	179.5	<0.001 *
	*Yes*	23	40	46.20		
	**Necrosis**					
	*No*	46	18.50	30.39	317	0.083
	*Yes*	19	22	39.32		
	**ER**					
	*Positive*	43	18	28.95	299	0.016 *
	*Negative*	22	23	40.91		
	**PR**					
	*Positive*	33	18	28.35	374.5	0.044 *
	*Negative*	32	24	37.80		

Note: ΤAΝs: Tumor-Associated Neutrophils; ER: estrogen; PR: progesterone. * *p* < 0.05 indicates statistical significance.

**Table 6:** Pearson χ^2^ test results for the relationship between the DR and the degree of differentiation (Grade).

		Degree of Differentiation (Grade)				
		Grade 1	Grade 2	Grade 3	χ^2^	*p*-Value
**DR**	Myxoid stroma	1 (14.3%)	8 (29.6%)	17 (54.8%)	9.448	0.051
	Keloid-like stroma	1 (14.3%)	10 (37%)	5 (16.1%)		
	Mature desmoplastic reaction	5 (71.4%)	9 (33.3%)	9 (29%)		

Note: DR: Desmoplastic reaction.

## Data Availability

The original contributions presented in this study are included in the article. Further inquiries can be directed to the corresponding author.
